# Is it prime time for carotid artery stenting in asymptomatic and symptomatic carotid artery stenosis?

**DOI:** 10.1186/s42155-026-00704-9

**Published:** 2026-05-30

**Authors:** Stefan Müller-Hülsbeck, Robert A. Morgan, Patrick Haage, Mohamad Hamady, Hicham Kobeiter, Romaric Loffroy, Florian Wolf, Gianpaolo Carrafielo, Matteo Stefanini

**Affiliations:** 1 Malteser Fördeklinikum St. Katharina, Flensburg, Germany; 2 St. George School, Middletown, USA; 3 HELIOS Universitätsklinikum Wuppertal, Wuppertal, Germany; 4 St. Mary Kings College: University of Aberdeen, Aberdeen, UK; 5 Hospital Henri Mondor, Créteil, France; 6 CHU Dijon Bourgogne, Dijon, France; 7 Medical University of Vienna, Vienna, Austria; 8 Università degli Studi di Milano, Milan, Italy; 9 Policlinico Casilino, Rome, Italy

Based on the outcomes of carotid artery stenting (CAS) from recent publications, the results for both symptomatic and asymptomatic stenoses are rather promising [1]. However, current treatment strategies after multi-disciplinary team decision-making are often limited to mainly symptomatic patients, who are treated with a scaffold, either in an acute situation for tandem lesion management during stroke therapy or in an elective setting [2].

Current Carotid Artery Stenting (CAS) guidelines recommend the procedure for patients with >70% symptomatic carotid stenosis or >70-90% asymptomatic stenosis, particularly for those at high surgical risk, often favouring patients <70 years old. However, the European stroke Organisation guideline on endarterectomy and stenting for carotid artery stenosis limits these recommendations to symptomatic lesions. When publishing these guidelines in 2021, they give the hint that outcomes after CAS versus CEA in patients with asymptomatic stenosis are anticipated in the near future [3]. This explains more or less that mainly symptomatic lesions are treated by CAS in daily practice.

With the recently published CREST-2 [4] data the management of these patients could change in institutions where endovascular specialists are available (e.g. interventional radiologist/neuroradiologist, angiologist /cardiologist or vascular surgeon with CAS expertise), where CAS could now be offered to asymptomatic patients as well. 

Several commentaries and editorials from different stakeholders involved in CAS provision have been published.

The American Heart Association published a commentary writing that stents but not endarterectomy reduced the stroke risk for asymptomatic CAS [5]. The findings of CREST-2 are similar to the SPACE-2 [6] and ECST-2 [7] trials and "conclude that there is no longer a role for routine carotid endarterectomy in persons with asymptomatic stenosis." As for CAS, the authors express caution for several reasons, including the small absolute difference between stenting and medical treatment alone and they suggest to advise these patients "to start intensive medical therapy immediately and to delay revascularization until ... symptoms develop." 

This statement is carefully balanced but is also restrictive; it implicates that future asymptomatic patients would not receive any revascularisation with CAS until they become symptomatic. This would be a considerable paradigm shift, as these patient groups would have been candidates for invasive surgical Carotid End-Atherectomy (CEA) in the pre-CREST-2 era.

An editorial from the Society of Vascular Surgery on CREST-2 concluded “that carotid interventions are perhaps the most widely studied of all medical procedures. Although CREST-2 certainly adds to this abundance of data, the results are not so overwhelming as to cast aside our wealth of accumulated knowledge regarding the benefits of BMT and carotid endarterectomy. The authors of the SVS editorial emphasize that the findings and recommendations in CREST-2 do not change the standard of care. The Society of Vascular Surgery agrees with the CREST-2 findings that medical management of all patients with carotid disease should be optimized and that TF (transfemoral) CAS procedures performed by highly trained, experienced interventionalists in carefully chosen patients may be an appropriate treatment. However, TCAR (Trans Cervical Artery Revascularisation) and CEA continue to play an important role in appropriately selected patients with asymptomatic carotid disease.”

A German commentary on CREST-2 also concludes with a statement that TCAR might play a role for the future in this patient group. The authors ended their commentary with the remark that CREST-2 did not include newer stent designs, e.g. dual-layer stents and their potential impact to achieve even better outcomes. [8]

Both commentaries from vascular surgery imply that stenting with new designs is very promising and could be performed via the transfemoral or transcervical access routes, by vascular surgeons, perhaps replacing TEA.

But why continue thinking about TCAR? From an IR’s perspective, it is not logical why suitable patients should undergo either TCAR or CEA. The exception to this is failed access to the carotid arteries via the aortic arch from the radial or femoral artery, justifying the use of CEA or TCAR.

The *European Society of Cardiology* released a blog on February 9, 2026 that low rates of disabling stroke were observed across all treatment groups, reinforcing the data of other recent interventional trials such as SPACE-2 and ECST-2. “The CREST-2 study challenges prior paradigms in the management of asymptomatic carotid-artery stenosis, as it constitutes the first randomized trial to present evidence in support of carotid-artery stenting over intensive medical management alone, reinforcing its role as a treatment option in this group of patients” (https://www.escardio.org/communities/councils/stroke/scientific-documents-and-publications/heart-and-stroke-blog/crest-2-new-evidence-in-asymptomatic-carotid-artery-stenosis-management/).

This statement is more or less echoed by one of the co-authors from the SPACE trials. With technological advances and growing neurointerventional expertise, carotid artery stenting has transitioned from an alternative option to a leading interventional strategy in carefully selected patients. CREST‑2 and related evidence herald a paradigm shift that must be reflected in upcoming guidelines and clinical decision-making. The evolving evidence supports moving from the traditional question “stenting or not?” towards the clinically more relevant consideration: “When and for whom is CAS the optimal treatment strategy?” [9] The CREST-2 evidence for the first time indicated that carotid stenting can lead to a net reduction in the risk of stroke. When CAS is added to intensive medical therapy, careful selection of patients, lesions, and operators will ensure the applicability of these findings [10]. 

CREST-2 further confirms that the procedural outcomes of CAS have progressively improved over time, narrowing the historical gap with surgery that was observed in earlier randomized trials.

However, an important aspect that deserves careful consideration is that the stents predominantly used in CREST-2 were first-generation, single-layer carotid stents. While these devices have proven to be effective, substantial technological advancements have occurred in recent years. Second-generation carotid stents, particularly mesh-covered or micromesh designs, have been specifically developed to address one of the major limitations of earlier devices: plaque prolapse and post-procedural embolization.

As highlighted by Musialek et al., modern micromesh-covered stents act not merely as scaffolding devices but as true cerebral protection systems, effectively isolating atherothrombotic plaque from the arterial lumen [11]. Multiple clinical and imaging studies have demonstrated a significant reduction in periprocedural and postprocedural cerebral embolic events with these devices, translating into improved neurological outcomes [1]. Therefore, it is reasonable to assume that the already favorable results observed in CREST-2 might further improve if contemporary second-generation stents were systematically adopted.

This consideration has important implications for future guideline recommendations and clinical practice. Current CIRSE guidelines emphasize that CAS outcomes are strongly dependent on appropriate patient selection, plaque morphology assessment, neuroprotection strategies, and device choice. [12] In this framework, asymptomatic patients with high-risk plaque features, long life expectancy, and low procedural risk may particularly benefit from CAS performed with modern devices in experienced centers. The CREST-2 data, interpreted through the lens of current technology, therefore support a potential expansion of CAS indications for asymptomatic patients rather than a restrictive approach.

In conclusion, CREST-2 represents a pivotal milestone for the future decision-making process for the ideal treatment of asymptomatic carotid artery stenoses. When integrated with contemporary device technology, CIRSE guideline recommendations, and real-world evidence supporting the superiority of high-volume high volume interventional practice, the CREST-2 trial reinforces the evolving role of CAS as a first-line treatment and primary option in shared decision-making for selected asymptomatic patients, when performed by rigorous trained physicians after careful anatomical selection [13]. Ideally, future research should focus on randomized evaluation of second-generation stents and on further refining patient selection algorithms, ensuring that the full potential of carotid artery stenting is realized in modern interventional radiology practice. Unfortunately, there is a high probability that these studies are unlikely to performed due to a lack financial funding.

Having CREST-2 data available for asymptomatic stenoses and looking retrospectively at landmark studies for symptomatic stenosis such as SPACE‑1 [14], CREST [15], EVA-3S [16] and ICSS [17], a clear trend becomes evident: Over the last decade, CAS outcomes have significantly improved. With these favorable data available, CAS should now be offered to our patients as the preferred treatment option. 

As we now have this up-to-date accumulated data in 2026 supporting CAS, future guidelines need to address this with CAS now established as a competitive first-line therapy in suitably selected patients.

What does that mean for endovascular specialists, especially interventional radiology and interventional neuroradiology? A bright future for stenting and minimal invasive treatment in experienced hands after careful patient selection can be predicted and IR should play a central role in offering these patient services as a clinical discipline. Do not be too shy, it is prime time for CAS! 

## Data Availability

Not applicable.
